# Cholera epidemic amidst the COVID-19 pandemic in Moroto district, Uganda: Hurdles and opportunities for control

**DOI:** 10.1371/journal.pgph.0000590

**Published:** 2022-10-12

**Authors:** Philip Orishaba, Marc Sam Opollo, Christine Nalwadda, Allan Muruta, Issa Makumbi, Kenneth Kabali, Anne Nakinsige, Phillip Lotee, Samuel I. Okware, Godfrey Bwire

**Affiliations:** 1 The Centre for Rapid Evidence Synthesis, College of Health Sciences, Makerere University, Kampala, Uganda; 2 Department of Integrated Epidemiology, Surveillance and Public Health Emergencies, Ministry of Health, Kampala, Uganda; 3 Department of Public Health, Faculty of Health Sciences, Lira University, Lira, Uganda; 4 Department of Community Health and Behavioral Sciences, Makerere University School of Public Health, Kampala, Uganda; 5 Emergency Operation Centre (EOC), Ministry of Health, Kampala, Uganda; 6 World Health Organization, Karamoja Regional Office, Moroto, Uganda; 7 Department of Health, Moroto District Local Government, Moroto, Uganda; 8 Uganda National Health Research Organization (UNHRO), Entebbe, Uganda; PLOS: Public Library of Science, UNITED STATES

## Abstract

**Introduction:**

On 21^st^ March 2020, the first COVID-19 case was detected in Uganda and a COVID-19 pandemic declared. On the same date, a nationwide lockdown was instituted in response to the pandemic. Subsequently, more cases were detected amongst the returning international travelers as the disease continued to spread across the country. On May 14^th^, 2020, a cholera epidemic was confirmed in Moroto district at a time when the district had registered several COVID-19 cases and was in lockdown. This study aimed to describe the cholera epidemic and response activities during the COVID-19 pandemic as well as the hurdles and opportunities for cholera control encountered during the response.

**Materials and methods:**

In a cross-sectional study design, we reviewed Moroto district’s weekly epidemiological records on cholera and COVID-19 from April to July 2020. We obtained additional information through a review of the outbreak investigation and control reports. Data were analyzed and presented in frequencies, proportions, attack rates, case fatality rates, graphs, and maps.

**Results:**

As of June 28^th^, 2020, 458 cases presenting with severe diarrhea and/or vomiting were line listed in Moroto district. The most affected age group was 15–30 years, 30.1% (138/458). The females, 59.0% [270/458], were the majority. The Case Fatality Rate (CFR) was 0.4% (2/458). Whereas home use of contaminated water following the vandalization of the only clean water source in Natapar Kocuc village, Moroto district, could have elicited the epidemic, implementing COVID-19 preventive and control measures presented some hurdles and opportunities for cholera control. The significant hurdles were observing the COVID-19 control measures such as social distancing, wearing of masks, and limited time in the community due to the need to observe curfew rules starting at 6.00 pm. The opportunities from COVID-19 measures complementary to cholera control measures included frequent hand washing, travel restrictions within the district & surrounding areas, and closure of markets.

**Conclusion:**

COVID-19 preventive and control measures such as social distancing, wearing of masks, and curfew rules may be a hurdle to cholera control whereas frequent hand washing, travel restrictions within the district & surrounding areas, and closure of markets may present opportunities for cholera control. Other settings experiencing concurrent cholera and COVID-19 outbreaks can borrow lessons from this study.

## Introduction

On 21^st^ March 2020, the first case of Coronavirus disease, 2019 (COVID-19) was detected in Uganda, the pandemic was declared in the country and a countrywide lockdown instituted [1]. In the days that followed, more COVID-19 cases were detected among returning international travelers at Entebbe International Airport. While COVID-19 Pandemic was spreading and Moroto district was under lockdown with several COVID-19 cases being confirmed, on May 14^th^, 2020, a cholera epidemic was confirmed in Moroto district, Northeastern Uganda.

Cholera, an acute diarrheal disease caused by ingestion of food and water contaminated with the bacterium *Vibrio cholerae*, remains of significant public health importance in developing countries such as Uganda [2, 3]. In developed countries, cholera incidence and mortality, and other diarrheal diseases have been eliminated by providing safe drinking water and maintaining proper sanitation and hygiene [4]. Globally, an estimated 1.3 to 4 million cases and 21,000 to 143,000 deaths due to cholera occur annually [5]. In most African countries, Uganda inclusive, improvements in Water Sanitation and Hygiene (WaSH) infrastructure particularly with regard to access to safe water and sanitation facility coverage is slow, resulting in suitable conditions for repeated cholera outbreaks [6–8]. Consequently, cholera outbreaks have previously occurred along the western border with the Democratic Republic of Congo (DRC), the Karamoja region to the north, and Kampala City slums [9]. A cholera outbreak last occurred in Moroto district in December 2015 [10].

Even though cholera has been labeled as a predictable, preventable, and treatable disease, outbreaks continue to spread across the country, with a high number of mortalities estimated to range from 61 to 182 deaths annually [9, 11]. In May 2020, a cholera epidemic was detected in Nadunget sub-county, rural part of Moroto district, Karamoja subregion. This cholera epidemic occurred when entire Uganda was under lockdown due to Corona Virus Disease 2019 (COVID-19) pandemic. Moroto district, like other districts in Uganda, was pre-occupied with observance of COVID 19 standard operating procedures such as restriction of movement, social distancing, curfew, hand washing, use of masks, and others. Whereas this was the first cholera outbreak recorded in Uganda while battling another infectious epidemic, countries like Ethiopia and Sudan both recorded cholera outbreaks during the COVID-19 era [12]. Such a cholera epidemic during the COVID-19 pandemic may pose significant challenges because as efforts are directed towards fighting the pandemic, the surveillance system as well as the health care system are paralyzed.

In Moroto district, whereas access to clean water is largely affected by issues related to climate, 73% of the population is reported to be located more than 30 minutes away from a water source. Furthermore, the use of improved sanitation facilities is as low as 11% in Moroto district [13]. With such low access to water, COVID-19 prevention and control guidelines may lead to a disruption of WASH services coinciding with restrictions of humanitarian services hence posing a great hindrance to campaigns against cholera epidemic and worsening the burden.

Henceforth, to interrupt the cholera epidemic and prevent spread within Moroto district, an epidemic was declared, and response actions initiated. This study aimed to describe the epidemic and the activities to control it amidst the COVID-19 pandemic in Moroto district, Uganda. In addition, the study examines the hurdles and opportunities encountered during the cholera epidemic control amidst the COVID-19 pandemic.

## Materials and methods

### Study design

This was a cross-sectional study involving a review of weekly epidemiological records and reports on cholera and COVID-19 in Moroto district from April 2020 to June 2020.

### Study settings

**Fig 1:**
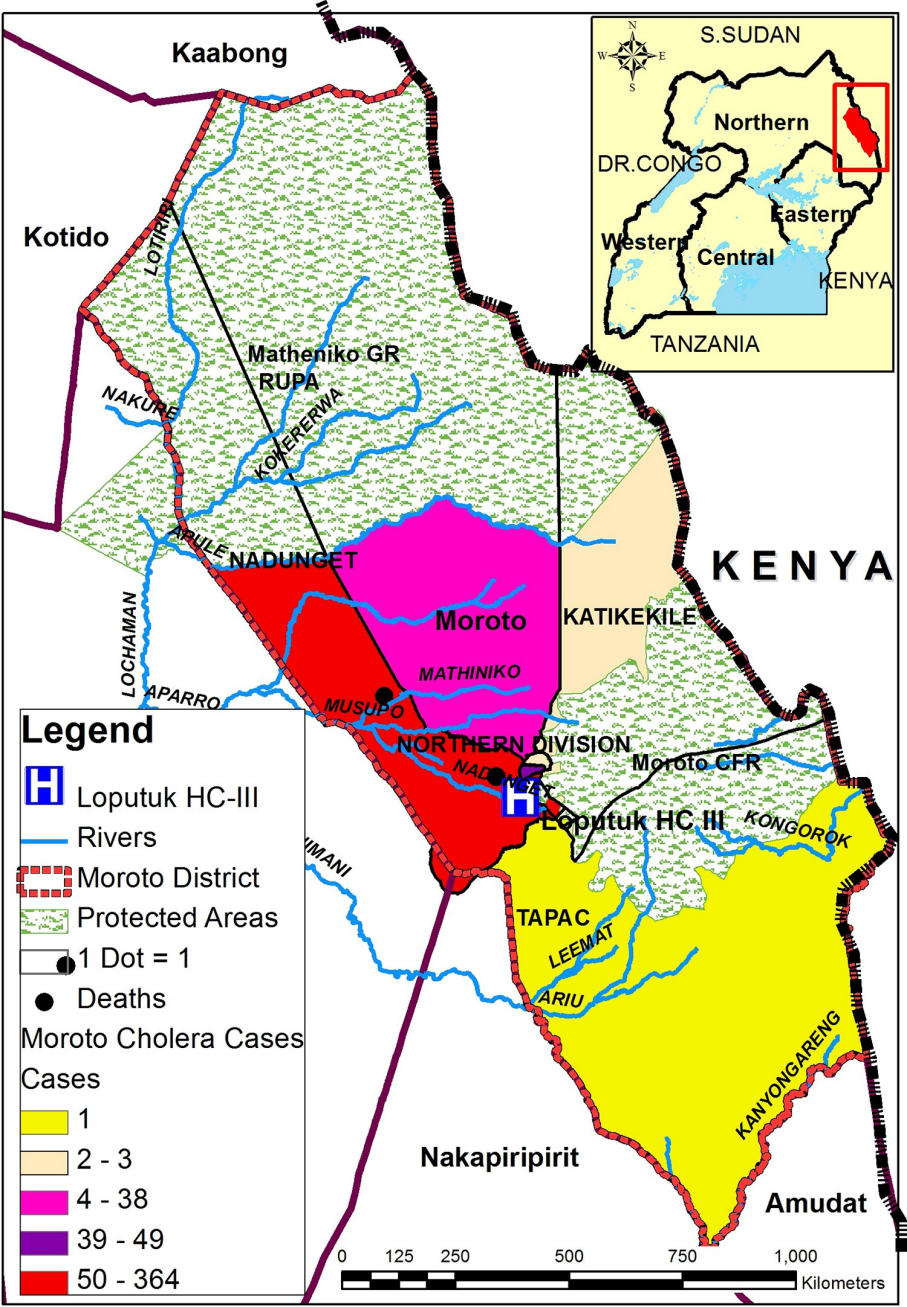
Map of Moroto district showing the cholera affected sub-counties and the location of Moroto district on the map of Uganda. The cholera affected sub-counties are in bright colors with each of the colors representing different number of reported cholera cases. Each of black dot represent 1 death. The shapefiles used to generate the map were from open access, the Humanitarian Data Exchange at https://data.humdata.org/.

Moroto district is one of the nine districts that make up the Karamoja sub-region in the northeastern part of Uganda. The district lies between latitudes 1°53’N, 3°05’N and longitudes 33°38’E, 34°56’E with a total area of 8,516 km^2^. The district is located about 434 kilometers by road, northeast of Kampala, Uganda’s capital. Location of Moroto district and administration units (**Fig 1**).

The population of Moroto district is 103,432 (UNCHS August 2014). The district is inhabited by the Karimojong, a distinctive agro-pastoral herder’s ethnic group which highly cherishes its traditions. Moroto district is characterized by little, scattered, and irregular rainfall resulting into patchy vegetation. Rainfall is in the range of 300 to 1200mm per year, with temperatures ranging from 20 Degree Celsius (°C) to 32°C.

Loputuk parish, where the outbreak was first reported, is located in Nadunget sub-county. Loputuk parish has 13 villages namely; Akwapua, Kambizi, Nachogorom, Natapar kocuc, Apetaoi, Kamera, Nadiket, Aworobu, Lokwakwa, Nangorikipi, Kalopwanya, Looi and Nasinyonoit. Similarly, the parish has 13 Local Council Ones (LCIs) and 26 Village Health Teams (VHTs). Karamojong live in *manyatas* (an enclosed residential area, surrounded by sharp thorns and small entry points for people and a larger entry point for cattle). One *manyata* has multiple families and a communal space for cattle. Natapar kocuc village in particular had 6 *manyatas* in total. Each *manyata* consisted of 10 to 15 households with 5 to 10 occupants in each household. The village dwellers are mostly nomads by nature and participate in trading local alcoholic brew named *Kwete*. The village members stay in small grass-thatched huts surrounding their livestock Kraal. Moroto District is only at 24.6% in hygiene levels, according to the District Water Officer, despite having 72% clean water coverage.

### Case definition used

According to the Ministry of Health (MOH) of Uganda Prevention and Control guidelines of Cholera of 2017, a case of cholera should be suspected in a patient aged five years or more, presenting with dehydration or death from acute watery diarrhea [14]. A case should also be considered a cholera suspect when they are aged two years or more with acute watery diarrhea in an area where a cholera epidemic has been declared [14]. A case of cholera is confirmed when *V*. *cholerae* serogroup O1 or O139 is isolated from any patient with diarrhea [14].

### Study variables

We obtained a line list including variables, namely, age of cases, sex, place of origin, date of onset of symptoms, treatment outcome (recovered or died), and presence of other medical conditions. The information extracted from the outbreak investigation reports included the status of home hygiene, water source, and availability of sanitation facilities.

### Inclusion and exclusion

We included; 1) cases meeting the standard case definition for cholera as per Uganda Ministry of Health cholera prevention guidelines and 2) seen for the period April to June 2020. We excluded 3 cases who reported a diarrhea lasting more than two weeks and one bloody stool case since this was likely due to other infectious conditions.

### Data collection, management and analysis

Patient quantitative data related to the epidemic was obtained from the Cholera Treatment Unit (CTU) manual case report forms that had been prepared in form of a line list. From the line list, data was abstracted using a pre-tested data abstraction form developed in EpiData Manager 4.6.2. The data was then exported to and analyzed in STATA 16.0. Data were analyzed and presented in frequencies, proportions, graphs and maps. Comparison of categories (age groups versus co-infection with malaria/ age versus sex) was made using the Chi-square test. We created maps using Arch GIS software licensed (ESRI, Redlands, California, United States of America). Shapefiles used to generate the map were from open access, the Humanitarian Data Exchange at https://data.humdata.org/. Data related to safe water, sanitation, and hygiene in the affected communities was collected through observation of the participants in their communities.

### Ethical clearance

This study used data collected as part of routine Ministry of Health Surveillance and outbreak work exempted from the Institution Review Board (IRB). The proposal to conduct the study was developed and submitted to the Uganda National Health Research Organization and obtained IRB exemption (*UNHRO/res/cholera/godf/17*.*05*.*2021*). No personal identifying information is shared.

## Results

### Description of the cholera epidemic

Starting April 29^th^, 2020, there had been cases of loose stool with vomiting and with/without a fever reporting to Loputuk Health Center III. Seven out of the eight fresh stool samples sent to the Central Public Health Laboratories tested positive for *V*. *cholera*, *Inaba* serotype in the weekly epidemiological report dated May 11^th^, 2020. The index case was a 17-year-old male patient from Natapar Kocuc village, Loputuk parish, Nadunget Sub County, who reported to the health center on April 29th, 2020, with acute watery diarrhea and severe dehydration. On May 4th, 2020, more cases with similar symptoms from the same parish as the index case also presented at Loputuk health center III. The health workers on duty that day suspected cholera. They reported to the Moroto District Health Officer, who requested fresh stool samples to be collected according to the MOH guidelines. The health workers sent the collected fresh stool samples to the Central Public Health Laboratories (CPHL) for laboratory analysis and confirmation of *Vibrio cholerae*. The Case Fatality Rate (CFR) in this epidemic was 0.4% (2/458). The two deaths were a 27-year-old male and a 34-year-old male, both from Kambizi village in Nadunget sub-county.

**Fig 2:**
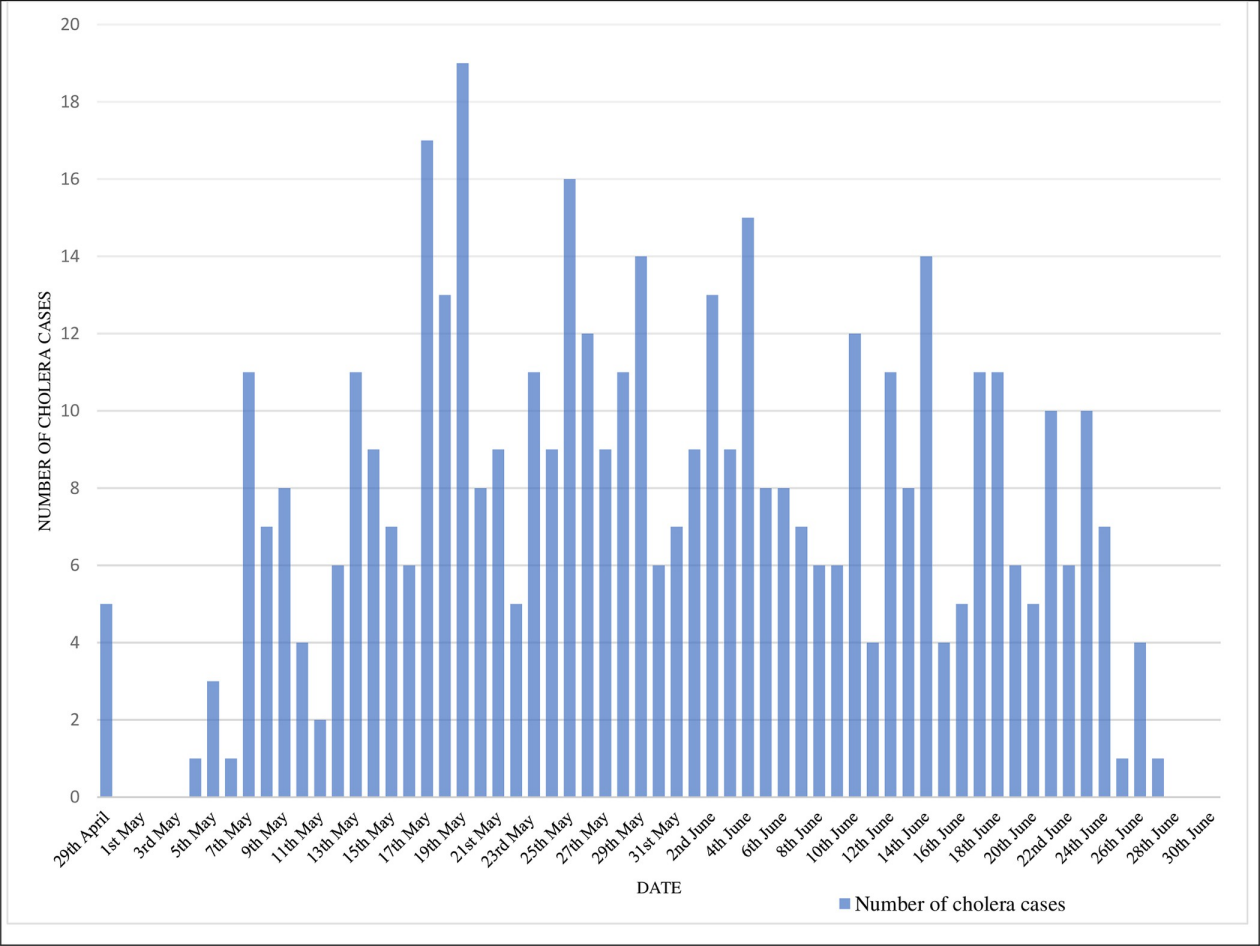
Daily reported new number of cholera cases in Moroto district during the period 29^th^ April-30^th^ June 2020. The outbreak lasted for approximately two months. Reactive oral cholera vaccination campaign was carried out when the outbreak was already on the declining trend.

The epidemic was characterized by different distinct epidemic waves showing a propagated or progressive epidemic pattern suggesting a person-to-person transmission with a peak at 19 cases on May 19th, 2020 (**Fig 2**).

Whereas all the patients (100%) presented to the health facility with severe acute diarrhea and vomiting, 94.8% (434/458) had a fever.

Most patients were female (59.0%) and aged 15–30 years (30.1%). More than 50% of the patients were also aged between 4 and 30 years. The age categories of the males were significantly different from those of females (P-value = 0.020). The proportion of females affected was significantly higher than that of males affected (59.0% versus 41.0%, P-value <0.001) (Table 1).

**Table 1:** Description of the study population.

Characteristic	Frequency (n = 458)	Percentage
**Age (years)**		
<1	15	3.3
1 to 4	102	22.3
5 to 14	105	22.9
15 to 30	138	30.1
31 to 59	62	13.5
≥60	36	7.9
**Sex**		
Female	270	59.0
Male	188	41.0

The attack rates were higher in females compared to males. All the age groups were affected, but the highest attack rate occurred among elderly persons. Nadunget sub-county had the highest attack rate at 9 persons per 1000. A total of 37 villages were affected, with Natapar Kocuc (33), Aworobu (19) and Kambizi (18) recording the most cases (Table 2). Loputuk parish had the highest attack rate at 30 cases per 1000 population, followed by Boma South with 8 cases per 1000 population and Lotirir with 6 cases per 1000 population. Parishes with no cases included Musupo, Narengenya, Lobuneit, Loyaraboth, Nakwanga, Natumukale, and Tapac.

**Table 2:** Cholera attack rates by age, sex, and sub-county at Loputuk Health Center III, Nadunget Sub County, Moroto district, April to June 2020.

Characteristic	Frequency (n = 458)	Population size (N = 103432)	Attack Rate (per 1000)
**Age (years)**			
<1	15	3273	4.6
1 to 4	102	15455	6.6
5 to 14	105	28500	3.7
15 to 30	138	25750	5.4
31 to 59	62	27652	2.2
≥60	36	2802	12.8
**Sex**			
Female	270	53686	5.0
Male	188	49746	3.8
**Sub-county**			
Nadunget	364	38836	9.4
South Division	49	9099	5.4
Rupa	38	25112	1.5
North Division	3	5097	0.6
Tapac	1	15441	0.1
Katikaikile	3	9847	0.3

Populations estimated using the UBOS’ Northern Region—Parish Level Profiles (Census 2014)—Last Updated on April 5th, 2019.

### Safe water, sanitation, and hygiene in the affected communities

**Fig 3:**
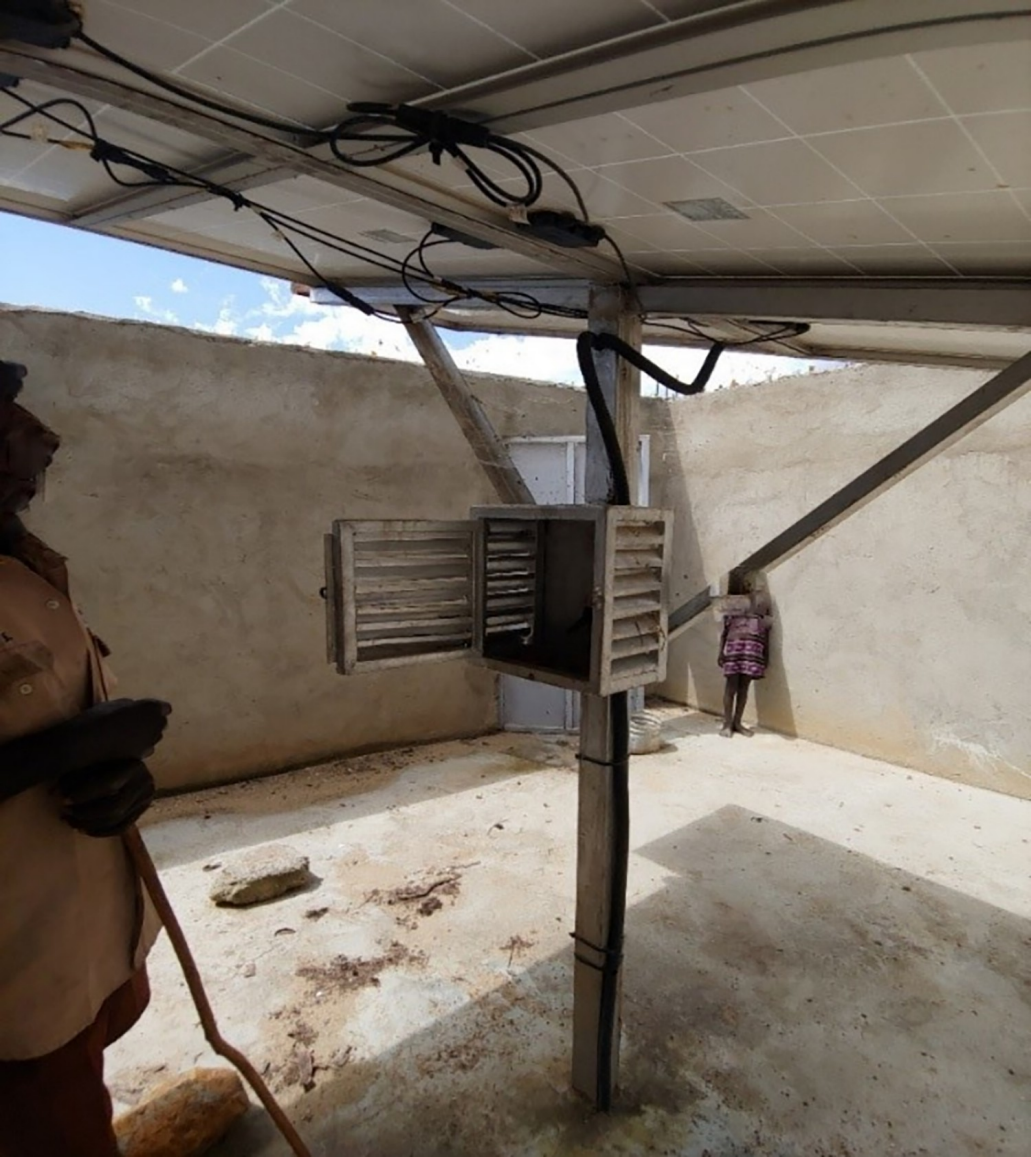
Shows the remains of the vandalized solar water pump station. The open metallic box is used to house the solar batteries and accessories (These items were vandalized).

Whereas in 2020, the district reports indicated a latrine coverage as low as 11% in Moroto district, there was evidence of massive open field defecation among community members in Natapar Kocuc village as well as at a new electrical station construction site which housed approximately 50 casual laborers. Regarding water sources, the village had two main water sources, one of which was a motorized solar borehole. Reports from the district office indicated that the borehole had been vandalized (battery and the inverter removed) approximately two weeks before the epidemic. The vandalized village borehole is shown in **Fig 3.**

**Fig 4:**
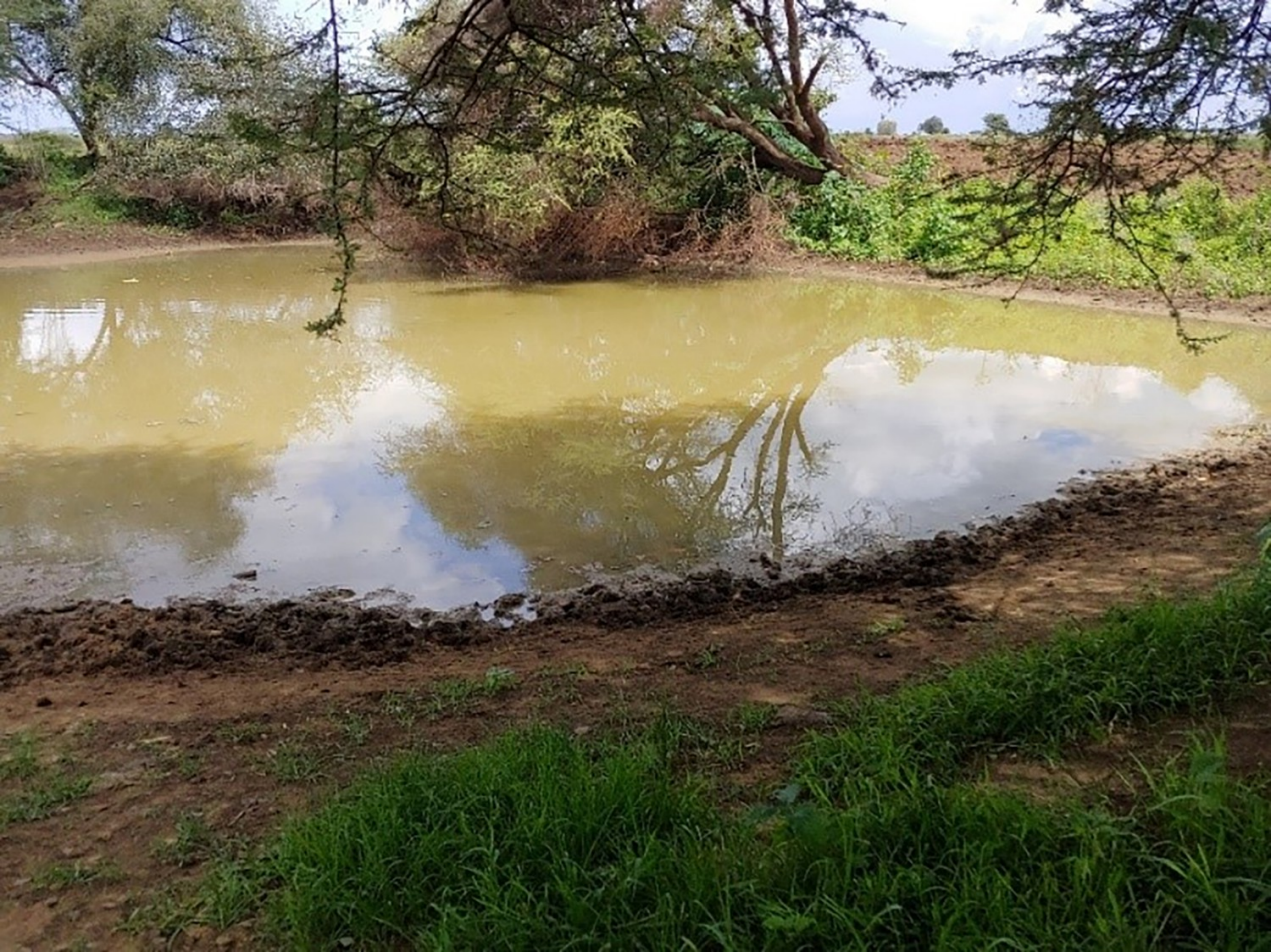
Major alternative water source that served Natapar Kocuc village and surrounding communities. This water source was meant to provide water for cattle but with no other near safe water sources the communities in the area had to share it with their animals. Note the water color and animal footsteps.

The second water source; an ordinary borehole had been nonfunctional for about two years. Without access to safe water nearby, the community members dug out a pond at the bed of a seasonal stream through which water from upstream ran whenever it rained. The dug-out pond that served the cholera affected population of Natapar Kocuc village is shown in **Fig 4.**

**Fig 5:**
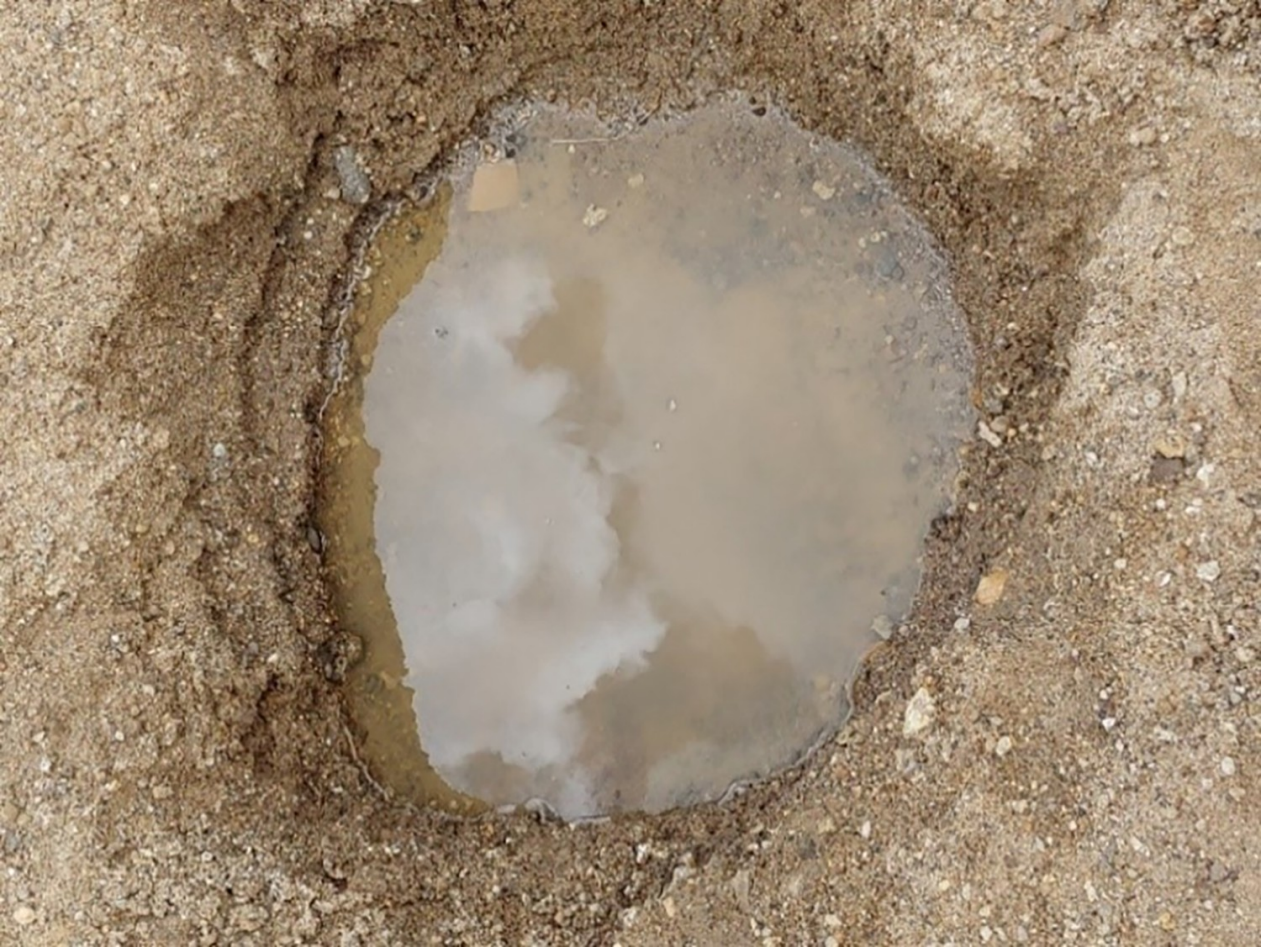
Best alternative water sources that served the community in Natapar Kocuc village following the vandalization of the only available safe water source in the village. A number of such small ponds were dug by the community along the bed of a seasonal stream. Note the dirty water and the water quantity.

In addition, some of the community members shared water sources with their domestic animals. One such alternative water source that was shared by the community in Natapar Kocuc village is shown in **Fig 5.** In Natapar Kocuc village, we observed several unhygienic practices at a food market where villagers vended local brew (*Kwete*) and boiled meat. Such unhygienic practices included touching money and then handling food with bare hands without first washing the hands, using dirty cutleries, sharing sedge grass straws as well as calabash pots, long nails of food handlers while others continued serving at the market even when they were not in good health.

### Oral Cholera Vaccination (OCV) campaigns

The response teams also conducted an oral cholera vaccination campaign employing a house-to-house vaccination strategy as well as a static point strategy for individuals that the team could easily access at their workstations. The team conducted the campaign between June and September 2020, in the five sub-counties affected by cholera in Moroto district. The vaccination campaign started with training health workers and sensitization of the general population starting on 7^th^ June 2020. The first dose was administered from 16^th^ to 21^st^ June 2020, while the second dose was administered from August 30^th^, 2020, to September 8^th^, 2020, for all persons aged one year and above. The teams were made up of health workers, VHT members & area Local Council chairpersons (LCIs) to mobilize community members. In addition to the VHTs and LCIs, central, district, sub-county, and parish supervisors supported the team. Alongside the vaccination exercise, the response teams maintained all other interventions on cholera response, including case management at the CTU, water, sanitation, hygiene promotion, and distribution of chlorination tablets.

An estimated population of 94,954 persons over the age of one year inhabiting the five sub-counties of Nadunget, Rupa, Katikekile, North Division, and South Division that had registered cholera cases was targeted. Out of a targeted **94,954** persons, **65,219** persons over one year (68.7%) received the vaccine. In addition to vaccination campaigns, the response team promoted/ enforced WASH practices, reinforced cholera surveillance, and conducted risk communication activities.

### Opportunities for cholera investigation and control

Several opportunities were noted during the outbreak response. The cholera epidemic occurred at a time when the Ministry of Health was implementing health promotion activities with respect to COVID-19 prevention and control standard operating procedures. The activities included airing messages such as encouraging the general public to wash their hands with soap and clean water frequently. In the same vein, organizations such as the UNICEF supported the district by providing water, sanitation, and hygiene (WASH) supplies like soap, detergents, hand sanitizers, chlorine, and handwashing stations for health facilities, families, and communities at risk.

Furthermore, at the time, all public gatherings had been banned and people in public facilities were cautioned to observe the recommended social distance, to frequently and appropriately practice hand hygiene (washing hands with soap as well as use of an alcohol-based hand rub, regularly cleaning and disinfecting surfaces, such as tables and door handles among others. These, among several other hygiene response activities are also relevant for cholera prevention and control.

There was restricted access to a number of public facilities such as disco halls, places of worship, academic institutions, cinema halls and sports centers. Additionally, there was restriction of travel to new areas whereby public and non-essential private transport was banned. With the curfew instituted as well, community members wouldn’t move from place to another after the stipulated movement time.

Furthermore, establishment of subcommittees to implement response activities was made possible by tapping on the already existing coordination framework of the COVID-19 response. As such, subcommittees that had already been established (community engagement & social protection, risk communication & social mobilization and leadership, stewardship, coordination & oversight) as part of the COVID-19 task force were the same committees tasked to respond to the cholera epidemic at the district level. Similarly, towards promoting awareness and advocacy on cholera and its recommended preventive measures, the community gatekeepers, traditional leaders, religious leaders and youth-led groups spreading messages on COVID-19 prevention and control used the same platform.

### Hurdles for cholera investigation and control

The significant hurdles observed in control of the epidemic included the COVID-19 measures that required health workers to keep social distances, frequent use of masks, and curfew. The cholera epidemic occurred when the COVID-19 pandemic had already strained the health system due to the need to reallocate some of the staff involved in general health service delivery to COVID-19 activities. There was a challenge continuing with essential service delivery and handling cholera cases while adhering to the COVID-19 preventive measures. Furthermore, the COVID restrictions, especially lockdown and curfew, meant that response teams worked for less time and returned early. Such restrictions disrupted WASH services as well as other humanitarian services that are key components of cholera prevention and control strategies. Other hurdles such as fragile socio-economic infrastructure, inability to actively mobilize resources for cholera control as well as logistical consequences were key barriers in the response. In addition to COVID-19, some suspected cholera cases had malaria as well. Such cases had to be tested for malaria using malaria Rapid Diagnostic kit Test (mRDT) and given treatment. The other significant hurdle was co-infection with malaria. Some of the cases with malaria had a severe form of cholera which required regular monitoring, yet the human resources for health were constrained or limited. Among the cholera cases, 27.1% (124/458) were diagnosed with malaria infection by mRDT. All age groups were affected by malaria infection, but the highest positivity occurred among children between 1 and 4 years (Table 3). Lastly, the epidemic occurred during the rainfall season. The heavy rainfalls towards the end of April and May exacerbated water contamination since open defecation was common.

**Table 3:** Malaria infection by age among suspected cholera cases at Loputuk Health Center III in Moroto district, April to June 2020.

	Malaria RDT	
Age (years)	Positive	Negative
<1	46.7 (7/15)	53.3 (8/15)
1 to 4	50.0 (51/102)	50.0 (51/102)
5 to 14	35.2 (37/105)	64.8 (68/105)
15 to 30	18.8 (26/138)	81.2 (112/138)
31 to 59	3.2 (2/62)	96.8 (60/62)
≥60	2.8 (1/36)	97.2 (35/36)

## Discussion

This study generated important lessons for controlling cholera in similar settings facing cholera and COVID-19 epidemics. Our study shows that it is possible to observe COVID-19 restrictive measures and successfully institute cholera control measures. For instance, the measures implemented during this response controlled the epidemic. Most importantly, some of the COVID-19 measures such as hand washing, restriction of big gatherings, travel restrictions between districts were complementary to the recommended cholera control measures in the national cholera guidelines [14]. Few studies have documented the concurrent cholera epidemic—COVID-19 pandemic situation and the use of reactive oral cholera vaccination campaigns. Given that COVID-19 is an ongoing pandemic, lessons from this study could be helpful to communities faced by both cholera and COVID-19 [15].

COVID-19 is known to disrupt social services [16], reducing access to health care and ultimately increasing mortality from other illnesses [17]. In this cholera epidemic during a COVID-19 pandemic, the case fatality rate (CFR) was lower than the World Health Organization recommended standard of less than 1%. We think this low CFR was due to COVID-19 measures such as increased surveillance and referral of severe cases complementing cholera control measures as a result ofhealth system alertness that facilitated notification, communication and fast return of results.

Although COVID-19 measures reinforced cholera control, some COVID-19 measures compromised the response activities. For example, to observe the curfew imposed to restrict COVID-19, the health workers deployed to sensitize communities and carry out contact tracing returned early from the field, reducing daily individual work output. This, in turn, could have contributed to the prolongation of the cholera epidemic, as was the case in Moroto. Our finding on the effect of the COVID-19 pandemic on cholera epidemic control is different from what was found by researchers in Nigeria, where the ongoing COVID-19 pandemic worsened the cholera situation [18]. The outbreak in Nigeria may have been much worse compared to our case in Moroto because the part of Nigeria where the cholera outbreak was reported was concurrently experiencing conflicts. Areas that experience conflicts and that are highly fragile break bonds between community members yet disunity in an area jeopardizes health care methods [19].

Another important finding was that the epidemic occurred in the community following the vandalization of the community safe water source. Therefore, the local authorities need to educate the communities to safeguard facilities/ equipment provided to them for the common good. The findings in this study which attribute the cholera epidemic to the home use of unsafe water, are also supported by the Uganda Ministry of Water and Environment performance report for 2020 [20]. The report indicated that the communities in the Karamoja region and Moroto district, in particular, had 40% of water sources contaminated with E. Coli [20]. Therefore for Uganda to meet the United Nation Sustainable Development Goal target for universal safe water coverage by 2030 [21], more efforts to increase access to safe water is required.

This study also documented several bad food handling practices in the market at Natapar Kocuc village that had potential to promote the spread of cholera and other foodborne infections within the community. Since the affected population were mostly semi-nomads, we anticipate that these bad practices were not localized to Natapar Kocuc village alone but also the entire Moroto district and other places visited by the semi-nomads. Therefore, in order to prevent such unhygienic practices ultimately result in infectious disease outbreaks [22, 23], the Moroto local government and the MOH will need to educate the food handlers on the safe food handling practices and also institute regular healthy fitness screening of the food handlers as well as hygiene inspections of all markets and eating places.

In addition to cholera and COVID-19, malaria infections were identified among the patients seen. These multiple infectious diseases can potentially worsen the poverty in the region through the reduction in production and diversion of resources for their treatment [24]. Thus, to address the multiple infections and malaria, the Ministry of Health will need to put more effort into better training of health care workers for appropriate diagnostic and management of patients with such co-infection in a pandemic context.

Even though a reactive oral cholera vaccination campaign was carried out during the outbreak, the decrease in the number of cases cannot be attributed to the vaccination exercise because it was evident that the outbreak was already on a declining trend at the time the first dose was administered. Much as the vaccination exercise not have been useful for controlling the outbreak, it can be useful for preventing future cholera outbreaks in the district.

This study was without some limitations. The findings shared were based on the daily line list kept at the CTU set up during response. As such, there may have been chance of non-, under or over reporting since we did not include the cases ourselves. However, since we cleaned the data and excluded the records that were not clear or had missing information, the data can support our conclusions. In addition, most of the authors also participated in the outbreak response. Therefore, they were able to identify errors in data and bring them to the attention of the broader team for the final decision.

## Conclusions

Home use of contaminated water and open defecation may have elicited the community-wide cholera epidemic in Moroto district amidst the COVID-19 pandemic. COVID-19 preventive and control measures such as social distancing, wearing of masks, and curfew rules may be a hurdle to cholera control whereas frequent hand washing, travel restrictions within the district & surrounding areas, and closure of markets may present opportunities for cholera control. The response team successfully implemented cholera control activities alongside COVID-19 standard guidelines. Other settings with concurrent cholera and COVID-19 like epidemics can borrow lessons from our study.
